# Lineage-specific proteins essential for endocytosis in trypanosomes

**DOI:** 10.1242/jcs.191478

**Published:** 2017-04-15

**Authors:** Paul T. Manna, Samson O. Obado, Cordula Boehm, Catarina Gadelha, Andrej Sali, Brian T. Chait, Michael P. Rout, Mark C. Field

**Affiliations:** 1 School of Life Sciences, University of Dundee, Dundee, Scotland DD1 5EH, UK; 2 The Rockefeller University, 1230 York Avenue, New York, NY 10021, USA; 3 School of Life Sciences, University of Nottingham, Nottingham NG2 7UH, UK; 4 California Institute for Quantitative Biosciences, University of California, San Francisco, CA 94158, USA

**Keywords:** *Trypanosoma*, Trafficking, Clathrin, Cytoskeleton, Flagellum, Evolution, Morphology

## Abstract

Clathrin-mediated endocytosis (CME) is the most evolutionarily ancient endocytic mechanism known, and in many lineages the sole mechanism for internalisation. Significantly, in mammalian cells CME is responsible for the vast bulk of endocytic flux and has likely undergone multiple adaptations to accommodate specific requirements by individual species. In African trypanosomes, we previously demonstrated that CME is independent of the AP-2 adaptor protein complex, that orthologues to many of the animal and fungal CME protein cohort are absent, and that a novel, trypanosome-restricted protein cohort interacts with clathrin and drives CME. Here, we used a novel cryomilling affinity isolation strategy to preserve transient low-affinity interactions, giving the most comprehensive trypanosome clathrin interactome to date. We identified the trypanosome AP-1 complex, *Trypanosoma brucei* (Tb)EpsinR, several endosomal SNAREs plus orthologues of SMAP and the AP-2 associated kinase AAK1 as interacting with clathrin. Novel lineage-specific proteins were identified, which we designate TbCAP80 and TbCAP141. Their depletion produced extensive defects in endocytosis and endomembrane system organisation, revealing a novel molecular pathway subtending an early-branching and highly divergent form of CME, which is conserved and likely functionally important across the kinetoplastid parasites.

## Abstract (precis)

**Summary:** Endocytosis is a vital process in most cells, and here we identify important proteins required for this process in trypanosomes. Significantly, these are unique and not present in animals, fungi or plants.

## INTRODUCTION

Eukaryotes originated from a lineage within the Thaumarchaeota, Aigarchaeota, Crenarchaeota and Korarchaeota (TACK) archaeal clade (Guy and Ettema, 2011; Raymann et al., 2015). Subsequent elaboration of the eukaryotic cell during eukaryogenesis gave rise to the nucleus, endomembrane system and acquisition of the mitochondrion (Martin et al., 2015). Following emergence of a true eukaryotic cell, the lineage rapidly diversified into multiple kingdoms or supergroups, represented for example, by plants, animals, fungi, amoeba and many protist lineages. The ∼1.5 billion year period since this radiation is vast, and while core metabolic and gene expression pathways are frequently well conserved, many cellular features have experienced extensive specialisations, in part as a response to adaptive evolutionary forces.

One aspect of this diversity that has received considerable attention is the endomembrane system, on account of this feature representing one of the more unique, yet flexible, aspects of eukaryotic cells. The primitive endomembrane system in the earliest eukaryotes gave rise to all of the endogenously derived internal compartments present in modern lineages (Schlacht et al., 2014). As a consequence of this extensive evolutionary history, compartments have experienced significant divergence, resulting in vastly different morphologies and functions, as reflected in diversification of the Golgi complex, lysosomes and endosomes. Furthermore, organelles unique to specific lineages such as the rhoptries of Apicomplexa and glycosomes of kinetoplastids are now known and are derived from endolysosomal ancestors and peroxisomes, respectively (Szöör et al., 2014; Tomavo, 2014). Residing within this compartmental variation are also apparently ancient and well-conserved molecular aspects of trafficking systems, for example the exocytic apparatus comprising the exocyst and endocytic pathways mediated by clathrin (Field et al., 2007; Koumandou et al., 2007).

One of the more diverse eukaryotic phyla are the Kinetoplastida, a group of flagellate protists, many of which are parasites. The African trypanosome *Trypanosoma brucei* lives and multiplies in the bloodstream and lymphatic system of its mammalian host, yet persists in the face of challenge from both the innate and adaptive immune systems (Brun et al., 2010). Immune evasion relies on multiple adaptations of the parasite cell surface composition and membrane-trafficking dynamics (Manna et al., 2014). The canonical defence of *T. brucei* is the expression of a dense coat of a single variant surface glycoprotein (VSG) which, through antigenic variation (i.e. the switching of expression between *VSG* genes), periodically creates an antigenically distinct cell surface (Schwede et al., 2015). As an additional defence, the VSG coat is constantly endocytosed and recycled, removing surface-bound immune effectors (Engstler et al., 2007; Field and Carrington, 2009). Perturbations of endocytic recycling severely limit parasite survival and infectivity *in vitro* and *in vivo* (Allen et al., 2003; Engstler et al., 2007; Natesan et al., 2011).

Common to all trypanosomatids is the polarisation of endocytosis and exocytosis to the flagellar pocket, a specialised plasma membrane invagination surrounding the base of the flagellum (Field and Carrington, 2009). This organelle is the gateway to and from the cell surface and essentially controls the host–parasite interface, but significantly also contributes towards interactions with multiple therapeutics (Alsford et al., 2013; Zoltner et al., 2015). Less elaborate, but similar structures, are present at the base of the cilium in other lineages, e.g. the proposed ciliary pocket in mammals and a ciliary partitioning system in *Tetrahymena* (Benmerah, 2013; Ounjai et al., 2013). It is unknown whether all of these flagellum-associated structures share common components and functionalities, and the potential that the flagellar pocket contains parasite-specific features limits the utility of comparative approaches that facilitated characterising much of the trypanosome endomembrane system (Field and Carrington, 2009). Recently the surface-exposed proteome of the trypanosome cell surface, the flagellar pocket and endomembrane system have been described (Gadelha et al., 2015; Shimogawa et al., 2015). While the precise definition and discrimination between these interconnected membrane systems is unclear, what has emerged is a remarkable level of novelty within the trypanosome surface membrane and endomembrane proteomes, with very few surface proteins demonstrating significant conservation across eukaryotes (Gadelha et al., 2015; Jackson et al., 2015). This extreme divergence potentially implies novel mechanisms of protein trafficking.

To directly explore the diversity of proteins associated with flagellar pocket function, we previously described affinity isolation of clathrin heavy chain [*T. brucei * (Tb)CHC] complexes using polyclonal antibodies, which identified several trypanosomatid-specific proteins that together mediating clathrin budding from the flagellar pocket membrane (Adung'a et al., 2013). However, owing to the weak and transient nature of many clathrin–partner interactions, the analysis was most probably under-sampled, and many interacting proteins were not isolated. Here, we applied a cryomilling method that better preserves protein–protein interactions while physically disrupting the robust sub-pellicular microtubule corset of the trypanosome cell (Obado et al., 2016a,b). We report an endocytic protein cohort encompassing the majority of the expected early endocytic proteins, together with several novel kinetoplastid-specific gene products. Two of this latter group, which we designate TbCAP80 and TbCAP141, control not only clathrin-mediated endocytosis (CME), but also the architecture and organisation of the broader endomembrane system. We suggest that these lineage-specific proteins are central to the coordination of membrane transport at the flagellar pocket, and are part of a growing list of proteins that mediate the control of function in this crucial organelle.

## RESULTS

### Cryomilling and mass spectrometry identifies novel clathrin-interacting proteins

A previous analysis of clathrin-interacting proteins in trypanosomes yielded a series of lineage-specific proteins we termed clathrin-associated proteins or CAPs. Several CAPs were characterised in some detail and demonstrated to mediate clathrin-budding events at the flagellar pocket membrane (Adung'a et al., 2013). However, many expected factors, including the adaptin AP-1 complex, were not retrieved despite being encoded in the trypanosome genome (Berriman et al., 2005). This suggests that additional proteins likely interact with clathrin, but were not captured by our earlier procedures, which used quite stringent conditions. Seeking a more complete view of the clathrin interactome, we harvested, flash froze and cryogenically lysed parasites by cryomilling them under liquid nitrogen to better preserve protein–protein interactions (Obado et al., 2016a,b). Insect- or bloodstream-form cells expressing CHC with a single allele tagged with a C-terminal GFP (CHC::GFP) were subjected to cryomilling. CHC::GFP was affinity captured under a range of differing salt and detergent conditions using polyclonal llama anti-GFP antibodies coupled to magnetic beads (Fridy et al., 2014).

**Fig. 1.**
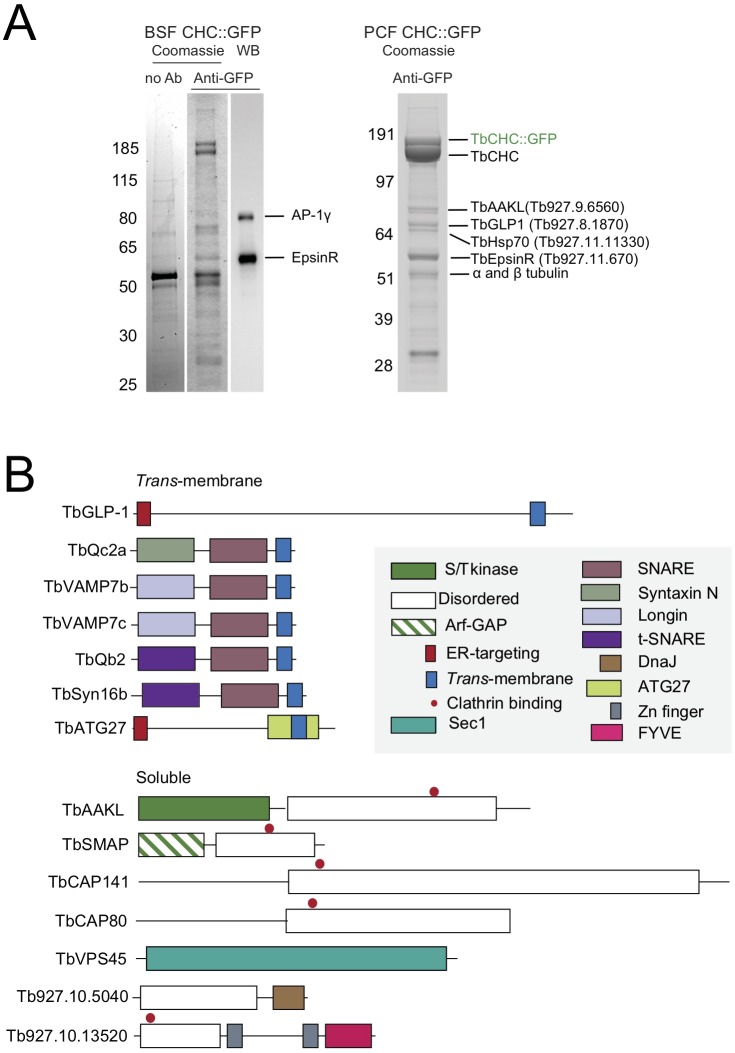
**Identification of *T. brucei* clathrin-associated proteins.** Clathrin and its associated proteins were immunoprecipitated from cryomilled powder generated from GFP-tagged CHC (CHC::GFP) bloodstream- or procyclic-stage cells. (A) Coomassie-stained SDS-PAGE shows bands specific to anti-GFP affinity isolates versus controls, confirmed as known CHC-associated proteins by western blot and MALDI-TOF MS. (BSF, bloodstream-form parasites; PCF, procyclic-form parasites) (B) Domain organisation of selected high-confidence clathrin-associated proteins identified by LC-ESI MS (see Table 1 and Table S1). Domain predictions were generated via HHPRED, HMMER and InterProScan.

Coomassie-stained SDS-PAGE of immunoprecipitates using optimised conditions and 50 mg of cell powder (∼5×10^8^ cell equivalents) suggested candidate interacting proteins present at substoichiometic levels, as expected (Fig. 1A). Mock immunoprecipitation performed with beads to which no antibody had been coupled served as the non-specific binding control. Western blotting and matrix-assisted laser desorption/ionisation time-of-flight mass spectrometry (MALDI-TOF MS) was used to verify the approach, and showed enrichment of known or predicted clathrin interactors (Adung'a et al., 2013; Borner et al., 2006, 2012, 2014; Pearse, 1976) (Fig. 1A). Larger scale immunoprecipitations using 300 mg of starting material for each condition were analysed by liquid chromatography-electrospray ionisation mass spectrometry (LC-ESI MS) and all identified proteins, from all conditions, are listed in Table S1. The top-ranked proteins, based upon a probabilistic log(e) score for correct protein identification combined with label-free quantification [exponentially modified protein abundance index (emPAI); Ishihama et al., 2005] from each condition were compared, and those proteins enriched in the anti-GFP antibody isolates versus the negative control selected (Table 1).

**Table 1.**
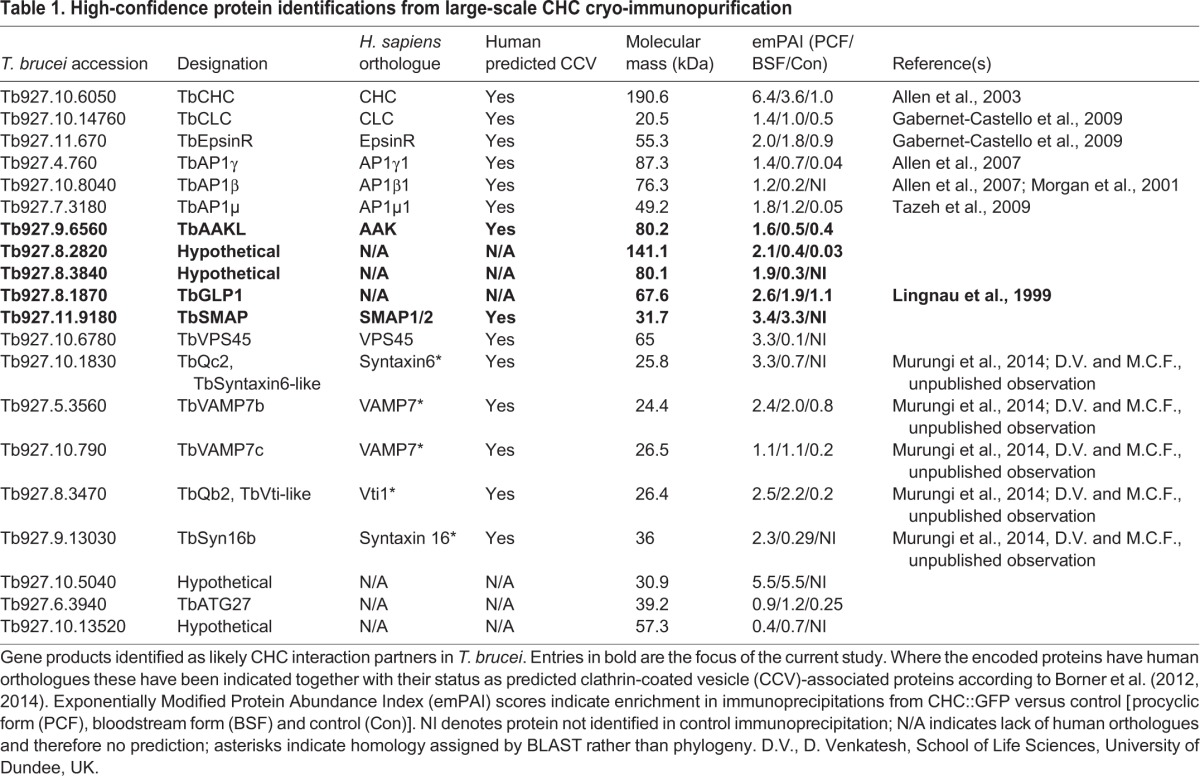
**High-confidence protein identifications from large-scale CHC cryo-immunopurification**

BLAST searches using the *T. brucei* strain 927 genome sequence database as queries were used to identify orthologues for conserved proteins, which were verified by reciprocal BLAST. As well as many known or predicted clathrin-interacting proteins, including subunits of the adaptor protein (AP)-1 complex and TbEpsinR (Allen et al., 2007; Gabernet-Castello et al., 2009; Tazeh et al., 2009), multiple partially characterised soluble N-ethylmaleimide-sensitive factor attachment protein receptor (SNARE) proteins (Murungi et al., 2014; Venkatesh et al., 2017) and proteins with no apparent homology to known human proteins were identified (Table 1).

Predicted domain architectures revealed several uncharacterised soluble cytosolic proteins, transmembrane-domain-containing SNARE proteins and the previously characterised trypanosomal Golgi-lysosomal glycoprotein 1 (TbGLP-1) (Fig. 1B) (Lingnau et al., 1999). In addition, TbEpsinR, Tb927.9.6560, Tb927.8.2820, Tb927.8.3840, Tb927.11.9180 and Tb927.10.13520 contain short, linear clathrin-binding motifs within predicted disordered regions (red dots in Fig. 1B), a common feature among many known clathrin-interacting proteins (Kirchhausen et al., 2014).

In light of these analyses, five candidate proteins were selected for further investigation. As we were predominantly interested in potential proteins that are cytoplasmic, soluble and associated with the flagellar pocket, we chose only a single integral membrane protein, TbGLP-1. The remaining four candidate proteins are predicted to be cytosolic with folded N-terminal domains and disordered C-termini containing predicted clathrin interaction motifs. Tb927.9.6560 has an N-terminal serine/threonine kinase domain and belongs to the same family of kinases as mammalian cyclin G-associated kinase (GAK) and AP-2 associated kinases (AAKs). These kinases are involved in clathrin-mediated trafficking processes through phosphorylation of the medium chains of the AP-1 and AP-2 adaptin complexes (Conner and Schmid, 2002; Ricotta et al., 2002; Umeda et al., 2000). This protein is hereafter referred to as *T. brucei* AAK-like (TbAAKL). Tb927.11.9180 (TbSMAP) has an N-terminal ArfGAP domain and belongs to the SMAP family of ArfGAPs implicated in clathrin-dependent vesicle transport (Tanabe et al., 2005; Schlacht et al., 2013). Of particular interest were two uncharacterised proteins with no apparent homology by BLAST to non-trypanosomatid proteins and no identifiable domains, Tb927.8.2820 and Tb927.8.3840 (hereafter TbCAP140 and TbCAP80, respectively).

### Localisation of putative clathrin-interacting proteins

**Fig. 2.**
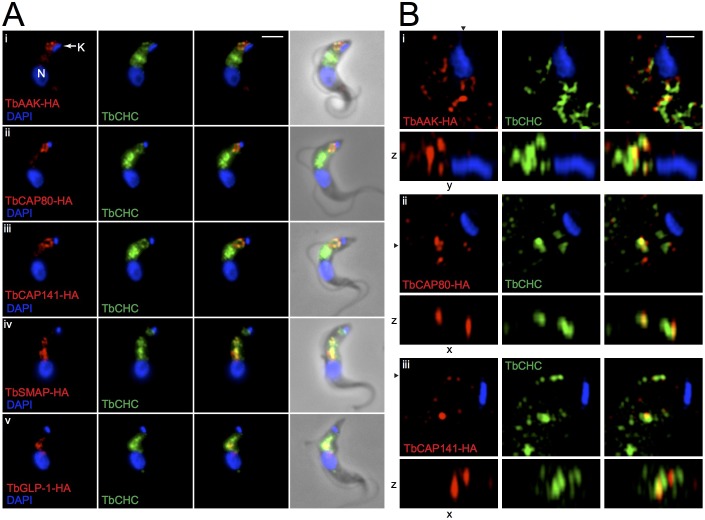
**Localisation of selected *T. brucei* clathrin-associated proteins.** C-terminal triple HA tags were added to selected clathrin-associated proteins at endogenous genomic loci via homologous recombination in bloodstream-stage parasites. (A) Immunofluorescence colocalisation showed correlation between clathrin-associated proteins (red, left panels) and CHC (green, central panels). Note that TbAAKL (i), TbCAP80 (ii) and TbCAP141 (iii) showed preferential colocalisation with clathrin punctae in the vicinity of the flagellar pocket. DAPI staining is in blue. N, nucleus; K, kinetoplast (indicated on top panel only). Scale bar: 2 μm. (B) Super-resolution SIM shows close association of clathrin-associated proteins (red, left panels, anti-HA antibody) with the flagellar pocket and clathrin-positive structures (green, centre panels, anti-TbCHC antibody) close to the kinetoplast. Boxes below the main panels show *z*-plane resections along the axis denoted by the black arrowhead for each image. Scale bar: 500 nm.

For immunolocalisation of candidate proteins in bloodstream-form trypanosomes, the lifecycle stage with the highest endocytic activity and the clearest dependence upon endocytic function for viability, PCR-generated DNA fragments encoding triple haemagglutinin tags were introduced into the endogenous genes via homologous recombination. C-terminal tagging was verified by western blotting and immunofluorescence. All five candidate proteins colocalised with clathrin, although to varying degrees. This variable colocalisation is to be expected given the diversity and functional segregation of inter-organelle membrane-trafficking events utilising the clathrin coat (Fig. 2A). An antibody to CHC widely labels the posterior region of the trypanosome cell, specifically between the two DAPI foci, the larger one representing the nuclear DNA and a second smaller one representing the mitochondrial DNA, the kinetoplast. This clathrin-rich region of the cell contains the single Golgi complex and almost the entirety of the endocytic compartments and, unsurprisingly, TbGLP-1 was observed here as multiple punctae. The distribution of TbSMAP closely resembled that of TbGLP-1, and both proteins colocalised extensively with CHC. The remaining three proteins were striking in their apparently restricted codistribution with some of the clathrin-containing structures. All three of TbAAKL, TbCAP80 and TbCAP141 colocalised with a distinct subset of clathrin punctae located close to the kinetoplast and therefore representing clathrin-coated structures at or near to the flagellar pocket membrane. Nascent clathrin-coated pits and vesicles are generally 100–200 nm in diameter, with several pits forming simultaneously at the flagellar pocket (Grünfelder et al., 2003). These clathrin structures are not readily resolved by conventional light microscopy, making it challenging to discern true associations with clathrin-coated membrane structures versus close apposition. We therefore employed structured illumination microscopy (SIM), which demonstrated a high level of colocalisation between all three candidate proteins and clathrin-positive structures (Fig. 2B; Movies 1–3). This is in contrast to a known exocytic protein TbSec15, which has been shown by SIM to form punctae interspersed with clathrin-positive structures at the flagellar pocket, but with essentially no colocalisation (Boehm et al., 2017).

### Sequence and phylogenetic analysis of *T. brucei* clathrin-associated proteins

**Fig. 3.**
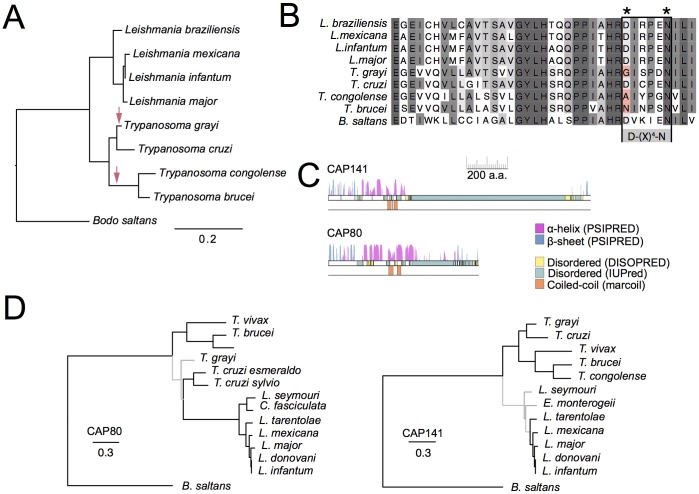
**Sequence and phylogenetic analysis of newly identified clathrin-associated proteins.** (A) Phylogenetic tree of the TbAAKL family across the kinetoplastids. Red arrows mark potential occurrences of mutations predicted to render the kinase inactive. (B) Sequence alignment showing putative inactivating mutations in kinetoplastid TbAAKL family kinases (asterisks). (C) Secondary structure predictions for TbCAP141 and TbCAP80. Note similarities with predicted extreme N-terminal β-rich regions followed by α-rich regions containing putative coiled coils and large disordered domains at the C-termini. (D) Phylogenetic analyses of TbCAP141 and TbCAP80 families showing distribution across the kinetoplastids. Trees shown are best-scoring maximum likelihood trees (PhyML). Black branches are supported >80, >80 or >0.99 (as assessed by RaxML, PhyML abd MrBayes, respectively), grey branches are below this level of support.

The evolutionary distribution of candidate genes was investigated to assess their potential relevance to the broader trypanosomatid lineage. The phylogenetic distribution of the SMAP family of ArfGAPs has recently been characterised in detail and demonstrated to be pan-eukaryotic (Schlacht et al., 2013). Hence, we focused on the remaining newly identified clathrin interactors, TbAAKL, TbCAP141 and TbCAP80. Orthologues of TbAAKL are found across the trypanosomatids and also in the sister group of free-living *Bodo saltans*, suggesting that the protein is conserved and an ancient aspect of the kinetoplastid membrane-trafficking system (Fig. 3A). However, in trypanosomes that lack the AP-2 complex and also the closely related AP-2-retaining *Tropidophorus* *grayi*, TbAAKL mutations within the conserved D-(x)_4_-N motif of the VIb subdomain are predicted to render the kinase inactive (Hanks and Hunter, 1995) (Fig. 3B). This suggests a possible relaxation of the requirement for AP-2-directed kinase activity in this lineage and, perhaps most significantly, because this mutation seems to predate the loss of the adaptor complex, it suggests the D-(x)_4_-N mutation could enable subsequent loss of AP-2.

**Fig. 4.**
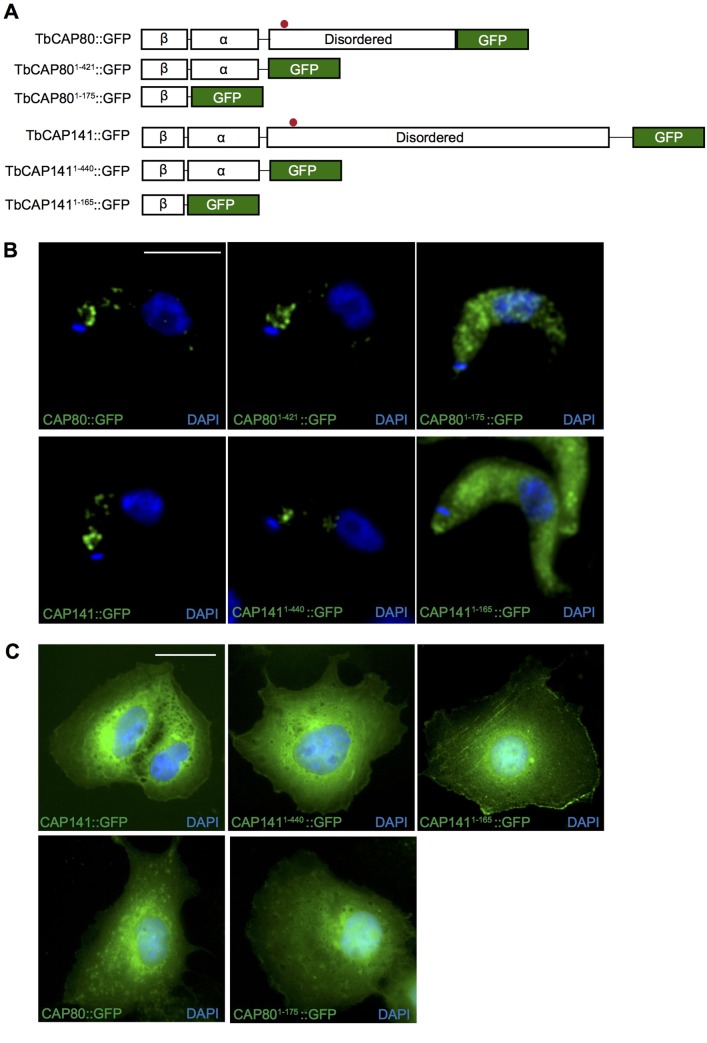
**Heterologous expression of TbCAP::GFP fusions.** (A) C-terminal GFP fusions were constructed for full-length TbCAP141 and TbCAP80, and truncated proteins bearing the entire predicted N-terminal globular domain and the predicted β-rich extreme N-terminus alone. (B) Expression of TbCAP full-length and truncation constructs in bloodstream-form parasites under the control of tetracycline. Low level expression necessitated amplification of native GFP fluorescence by staining with anti-GFP followed by fluorescent secondary antibodies. Scale bar: 5 μm. (C) Expression of TbCAP full-length and truncation constructs in COS-7 cells. Scale bar: 20 μm.

Because TbCAP80 and TbCAP141 were completely uncharacterised, we first examined their predicted secondary structures. Unexpectedly, these were strikingly similar despite no apparent sequence similarity, consisting of an extreme N-terminal that is predominantly β-sheet containing, followed by a relatively large predominantly α-helical region containing putative coiled-coils (Fig. 3C). Downstream of the folded N-terminal domain, both TbCAP80 and TbCAP141 are predicted to contain a large disordered region. Domain prediction tools (HHpred, HMMER and InterproScan) returned no high-confidence predictions, although there is very weak support for the extreme N-terminal β-region representing a degenerate C2 domain. Furthermore, clathrin-binding motifs are located within the disordered regions of both TbCAP80 and TbCAP141. Both are found as single copy genes across the trypanosomatids and also in the sister group Bodonidae, but neither protein has identifiable orthologues outside of the Discicristata (Fig. 3D). Significantly, this overall predicted architecture for TbCAP80 and TbCAP141 is reminiscent of several characterised monomeric clathrin adaptors in which the N-terminal region is a membrane-binding domain while the C-terminus appears flexible and is predicted to be disordered; examples include the ENTH/ANTH domain epsin, epsinR and calmodulin (CALM) family proteins, suggesting a general architectural conservation consistent with clathrin binding (Kirchhausen et al., 2014). For functional assessment of CAP141 and CAP80 domains, full-length and truncated forms were cloned into vectors for expression in bloodstream-stage trypanosomes and mammalian cells (Fig. 4A) (Sunter et al., 2012). In bloodstream-form trypanosomes, full-length CAP141 and CAP80 GFP fusions localised to punctae at or near to the flagellar pocket, as was seen for the endogenously tagged proteins. However, this flagellar pocket localisation was apparently readily saturable, likely reflecting a limited number of available binding sites, with higher expression levels leading to mis-localisation when compared to endogenously expressed fusion proteins (Fig. 4B; Fig. S1). Removal of the predicted disordered domains, containing the putative clathrin-binding motifs, from either protein had no apparent effect upon the flagellar pocket localisation, whereas expression of only the extreme N-terminal β-rich region as a GFP fusion led to a loss of flagellar pocket targeting (Fig. 4B).

In a further attempt to determine whether the predicted architectures of TbCAP80 and TbCAP141 functionally correspond to an N-terminal membrane-addressing domain and potential recognition of specific lipid species at the membrane, we expressed TbCAP80 and TbCAP141 as GFP fusion proteins in COS7 cells (Fig. 4A). This approach was chosen as expression in the mammalian cell background presumably isolates each protein from trypanosome interaction partners that may influence localisation as well as representing a better-characterised membrane system, where the localisations of specific phosphoinositide species are well documented. Full-length TbCAP141 had a largely perinuclear and reticular distribution (Fig. 4C), although with some clear plasma membrane association. The internal localisation for TbCAP141 may be a result of overexpression as seen in the homologous system. The trypanosome expression studies suggest that the large disordered region of this protein is likely dispensable for membrane localisation. Therefore, a truncated version of TbCAP141, encoding only the N-terminal predicted folded domain (residues 1–440) fused to GFP, was expressed. This construct showed a much more diffuse cytosolic signal while also retaining plasma membrane association (Fig. 4C). Finally, we expressed the extreme N-terminus of TbCAP141, corresponding to the potential C2-like fold (residues 1–165), as a GFP fusion. This construct failed to localise to the flagellar pocket in bloodstream-form trypanosomes but showed the most pronounced plasma membrane localisation, suggesting that the N-terminal β-rich domain contains a membrane localisation signal (Fig. 4C). We note, however, that in the absence of a full understanding of the lipid environment at the trypanosome flagellar pocket these data are merely supportive of this conclusion.

In contrast to TbCAP141, TbCAP80 constructs encoding both full-length protein and the extreme N-terminal β-rich domain fused to GFP were largely cytosolic and lacked clear membrane association (Fig. 4C). This suggests that the targeting information required for localisation of TbCAP141 to the plasma membrane, or the flagellar pocket membrane in the native trypanosome cell, is conserved across eukaryotes, whereas TbCAP80 likely relies upon a trypanosome-specific factor as an accessory determinant.

### TbSMAP, TbCAP80 and TbCAP141 are essential for endocytosis

Identification via CHC-GFP immunoprecipitation and colocalisation with clathrin-containing structures suggest endocytic functions for TbAAKL, TbSMAP, TbCAP80 and TbCAP141. Many characterised endocytic proteins in *T. brucei* are essential for viability in bloodstream-form trypanosomes, as is endocytosis itself, suggesting that if TbAAKL, TbSMAP, TbCAP80 and TbCAP141 are indeed major players in endocytic trafficking, they should also have a significant impact on proliferation and viability.

**Fig. 5.**
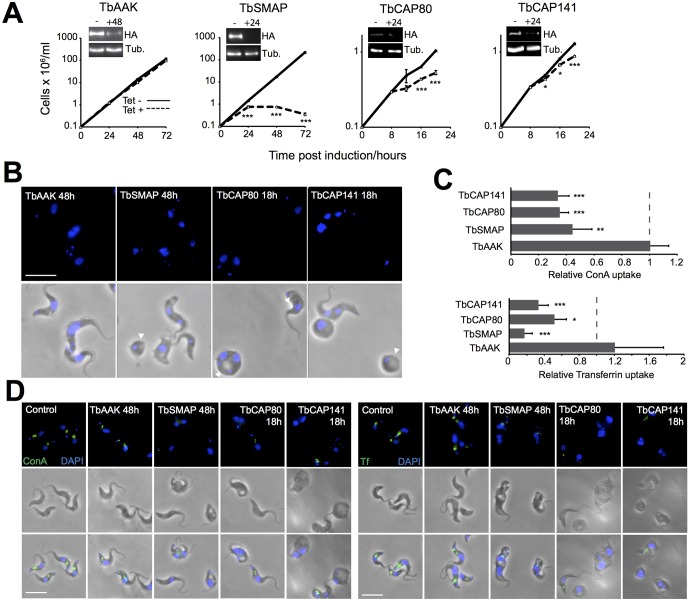
**Phenotypic consequences of clathrin-associated protein depletion.** Tetracycline-inducible RNAi cell lines for selected clathrin-associated proteins were generated in HA-tagged bloodstream-form cells. (A) Effects of tetracycline addition on protein levels as assessed by western blotting before RNAi induction and 24 h after induction using an anti-HA antibody (insets, - and +24, respectively), and its effect on cell proliferation expressed as mean±s.e.m. from three independent repeats. – (dashed lines), uninduced controls. (B) Morphology of induced RNAi cells at the indicated time points. Blue, DAPI staining; white arrowheads denote vacuolar structures viewed under phase-contrast conditions that are reminiscent of swollen flagellar pockets. (C,D) Effects of clathrin-associated protein depletion on uptake of FITC–transferrin or FITC–ConA. (C) Quantification of FITC–transferrin or FITC–ConA uptake in induced RNAi cells versus uninduced controls. Data are mean±s.d. of at least 50 cells per condition from two independent experiments normalised to non-induced controls. (D) Representative images showing FITC–transferrin or FITC–ConA (green) accumulation following a 45-min pulse. Blue, DAPI staining. Scale bars: 5 μm. *P<0.05, **P<0.005, ***P<0.001 versus control (*t*-test).

To address these questions, we generated inducible RNAi cell lines in endogenous-locus-tagged bloodstream-form backgrounds for each candidate protein. As for the localisation studies, we have focused on bloodstream-form parasites as this life cycle stage has the highest endocytic activity as well as the clearest link between endocytic activity and viability. Following RNAi induction, protein depletion was assayed by western blotting and cell proliferation was recorded (Fig. 5A). TbSMAP depletion led to a proliferative inhibition after ∼24 h. Depletion of either TbCAP80 or TbCAP141 produced strong proliferative inhibition from as little as 12 h post induction, which is consistent with previous whole-genome studies (Alsford et al., 2011). By contrast, despite strong protein depletion, silencing of TbAAKL had no obvious effect upon parasite proliferation. Furthermore, the proliferative defects following TbSMAP, TbCAP80 or TbCAP141 depletion were accompanied by morphological abnormalities (Fig. 5B). In particular, cells became round and this was frequently accompanied by flagellar pocket swelling, with a large vacuolar structure resulting from failure of CME at the flagellar pocket seen under phase-contrast conditions, although this can also potentially arise from an impact on many trafficking events (Adung'a et al., 2013; Allen et al., 2003; Spitznagel et al., 2010). These morphological effects were absent in TbAAKL-depleted cells, but more severe and faster in onset following TbCAP80 and TbCAP141 depletion compared to upon depletion of TbSMAP.

As the enlarged flagellar pocket phenotype of TbSMAP-, TbCAP80- or TbCAP141-depleted cells suggested that there was endocytic inhibition, we assayed this directly by following the uptake of fluorescently labelled probes. Transferrin is selectively acquired via the transferrin receptor (Steverding, 2000) whereas the lectin concanavalin A (ConA) is a non-selective binder of mannose-containing cell surface glyocproteins, predominantly VSG in the trypanosome (Allen et al., 2003). When compared to non-induced control cells, the uptake of both ligands over a 45 min period was strongly inhibited, following depletion of TbSMAP, TbCAP80 and TbCAP141 (Fig. 5C,D). Consistent with this absence of a proliferative or morphological phenotype, TbAAKL depletion had no apparent impact upon endocytic cargo uptake, indicating that TbAAKL is not required for maintaining endocytic transport.

### TbCAP141 and TbCAP80 are required for multiple aspects of flagellar pocket function

The effects of depleting several endocytic proteins are well characterised at the ultrastructural level, and typically lead to enlargement of the flagellar pocket membrane area and luminal volume, together with additional changes associated with delivery or removal of membrane carriers to or from the cell surface (Adung'a et al., 2013; Allen et al., 2003; García-Salcedo et al., 2004; Manna et al., 2015).

**Fig. 6.**
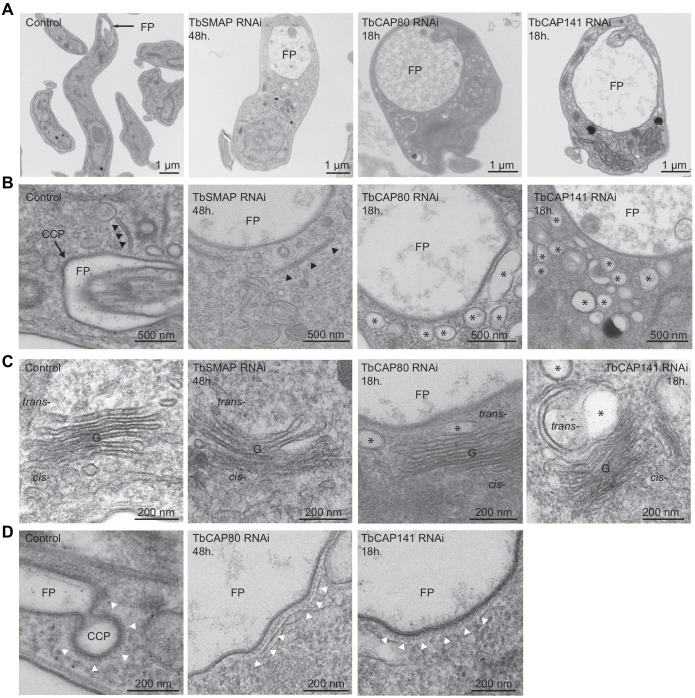
**Effects of clathrin-associated protein depletion on endomembrane system ultrastructure.** Representative transmission electron micrographs of ultrathin resin sections of bloodstream-form parasites after RNAi-mediated depletion of clathrin-associated proteins. (A) Gross ultrastructural defects, in particular swelling of the flagellar pocket (FP) and cytoplasmic vacuolisation. (B) Tubular endosomes (black arrowheads) are apparent in the vicinity of the flagellar pocket of control and TbSMAP-depleted cells. A clathrin-coated pit (CCP) profile is seen at the flagellar pocket membrane of the control section (black arrowheads). Extensive vacuolisation (asterisks) is apparent in both TbCAP80- and TbCAP141-depleted cells. (C) Golgi (G) profiles. The control cell shows numerous ER–Golgi transport intermediates. TbSMAP depletion has little apparent effect upon Golgi ultrastructure, whereas depletion of TbCAP80 or TbCAP141 causes vacuolisation and swelling (asterisks) of the *trans*-cisternae. (D) Clathrin-coated profiles (white arrowheads) of control and TbCAP80- or TbCAP141-depleted cells. Aberrant large and flat coated profiles are seen in TbCAP80- or TbCAP141-depleted cells.

We therefore prepared ultrathin sections of bloodstream-form cells depleted of TbSMAP, TbCAP80 and TbCAP141 by fast isothermal fixation for transmission electron microscopy (TEM). Control cells show the polarised distribution of the *T. brucei* endocytic system, with the posterior flagellar pocket often associated with clathrin-coated pits and vesicles (Fig. 6A). The vacuoles that are seen under phase-contrast light, when observed by conventional microscopy in TbSMAP-, TbCAP80- and TbCAP141- depleted cells (Fig. 5), can be confirmed to represent swelling of the flagellar pocket (Fig. 6A). While TbSMAP-depleted cells displayed normal endosome and Golgi complex profiles with clear tubular sorting endosomes present (Fig. 6B,C), extensive vacuolisation of the cytoplasm was seen in cells depleted of either TbCAP80 or TbCAP141 (Fig. 6B). These swollen vacuolar structures were prominent in the vicinity of the flagellar pocket and also associated with the *trans* face of the Golgi stack (Fig. 6C) indicating more widespread endomembrane system defects. Finally, we examined the morphology of clathrin-coated structures associated with the flagellar pocket. Control cells displayed many clear clathrin structures, ranging from early, shallow pits to deeply invaginated and pre-scission necked pits (Fig. 6D). No such structures could be identified in TbSMAP-depleted cells, whereas TbCAP80- and TbCAP141-depleted cells formed extended shallow pits. This suggests that the endocytic defects in these cells arise from a failure of curvature generation and coated pit progression. Depletion of the characterised clathrin adaptor proteins TbCALM and TbEpsinR produces a similar phenotype (Manna et al., 2015), supporting common and non-redundant functions for these proteins. Although of uncertain significance, we also note that in TbCAP80- and TbCAP141-depleted cells, the enlarged flagellar pockets were occasionally (2 of 28 thin sections, 1 of 20 thin sections, respectively) associated with subpellicular microtubules, which are a non-canonical feature of this specialised membrane domain, likely due to loss of differentiation from the plasma membrane (Fig. S2). Furthermore, depletion of either TbCAP80 or TbCAP141 also led to the appearance of cytoplasmic axonemes, often associated with a paraflagellar rod (3 of 28 and 5 of 20 thin sections, respectively) (Fig. S2). Although this observation could suggest a loss of cell polarity, and has been observed upon knockdown of functionally related proteins (Price et al., 2007), this phenotype has also been observed to be a consequence of ablation of proteins with functions apparently unrelated to those studied here (Morgan et al., 2005; Baines and Gull, 2008; Morriswood and Schmidt, 2015; Sheader et al., 2005). In summary, the most representative ultrastructural defects observed suggest crucial roles for TbCAP80 and TbCAP141 in both endocytosis and Golgi complex and flagellar pocket function and are notable by their extensive scope, impacting a considerable proportion of the endomembrane system.

## DISCUSSION

**Fig. 7.**
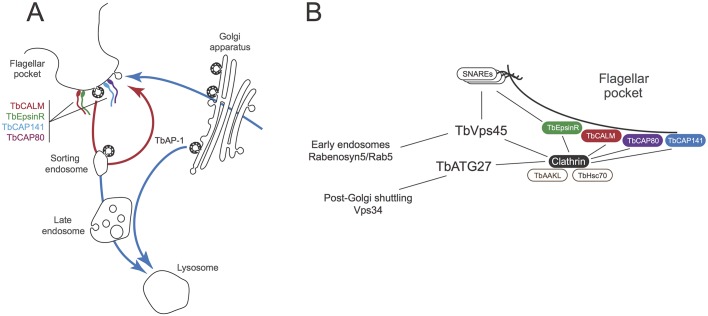
**Interactome** **subtending CME** **in trypanosomes.** Whereas clathrin is distributed widely throughout the trypanosome endomembrane system, flagellar pocket-specific factors act in the earliest stages of endocytosis. TbCALM and TbEpsinR are likely recruited via localised lipid accumulation. TbCAP141 and TbCAP80 are also enriched in flagellar pocket-associated clathrin structures. We propose that the large disordered regions of these proteins likely contribute to the generation of local membrane curvature through molecular crowding. Additionally, a conserved core of clathrin-associated accessory proteins (TbVps45, TbAAKL and TbHsc70) likely act to coordinate clathrin coat dynamics and regulate the sorting of the conserved SNARE machinery.

Trypanosomes likely diverged over one billion years ago and many examples of extreme specialisations within membrane trafficking, gene expression and nuclear organisation in these organisms are known (e.g. Daniels et al., 2010; DuBois et al., 2012; Field and Carrington, 2009; Obado et al., 2016b). Comparative genomics demonstrates that trypanosome endocytosis is divergent (Adung'a et al., 2013; Field et al., 2007; Manna et al., 2013, 2015), based both on the absence of orthologues of multiple endocytic genes that are present in animals and fungi, and recent identification of several lineage-specific proteins that are associated with clathrin. We have further investigated the clathrin interactome as our earlier study failed to identify many expected and conserved gene products. By performing cryomilling and improved affinity purification, we indeed identified many of these anticipated interaction partners (Fig. 7) and more lineage-specific proteins, expanding the evidence that novel mechanisms are operating in these cells.

Specifically, we recovered all subunits of AP-1, many SNAREs expected to be associated with the endosomal/post-Golgi trafficking systems, trypanosome orthologues of AAK and SMAP, as well as Hsc70 and TbEpsinR, with these latter two also being identified in our earlier work (Adung'a et al., 2013). We also recovered Vps45, the endosomal Sec1/SM protein, which in higher eukaryotes interacts with multiple SNAREs and which, therefore, may well have acted as a bridge between clathrin and the SNARE proteins recovered in this screen. Vps45 also associates with Rabenosyn5 in mammalian cells, providing an additional connection to early endosomes. The SNAREs in particular are likely associated with endosomal and recycling trafficking based on recent phylogenetics and immunoisolation investigations (Venkatesh et al., 2017). We also recovered TbGLP-1, an abundant trypanosome-specific transmembrane domain protein associated with the Golgi complex (Lingnau et al., 1999). The identification of several transmembrane domain proteins, together with AP-1 and Vps45, gives us confidence that we have now sampled the trypanosome endosomal machinery to a considerable depth, as does the observation that a very similar cohort of CME proteins are present in *T. cruzi* (Kalb et al., 2016). It is of interest that we detect AP-1 and not any other adaptin complex proteins, and it is likely that AP-1 is involved in pathways delivering multiple proteins to the lysosome, but not directly in endocytosis from the plasma membrane (Allen et al., 2007; Morgan et al., 2001; Zoltner et al., 2015). The trypanosome orthologue of the autophagy protein Atg27 is also of interest; this protein lacks a mammalian homologue but has been characterised in yeast where it cycles between the Golgi and peripheral membrane structures, and controls trafficking of another autophagy protein Atg9 (Backues et al., 2015; Yen et al., 2007).

The current study, and recent publications from our laboratory and others, allow us to assemble a model for a protein interaction network underlying flagellar pocket endocytic function (Fig. 7) (Adung'a et al., 2013; Demmel et al., 2014; Gabernet-Castello et al., 2009; Manna et al., 2015). We suggest that the local generation of a phosphoinositide signal, likely phosphatidylinositol 4,5-bisphosphate [PI(4,5)P_2_], is an essential step in clathrin-coated pit formation, and this signal is sensed by the phosphoinositide-binding clathrin adaptors TbEpsinR and TbCALM, and possibly TbCAP80 and TbCAP141 (Demmel et al., 2014; Manna and Field, 2016; Manna et al., 2015). *T. brucei* homologues of Hsc70 and GAK/AAK family kinases are also associated with clathrin structures but their roles remain unclear due to apparently minor phenotypes following RNAi-mediated depletion.

TbCAP80 and TbCAP141 have essential roles in CME; that these apparently abundant proteins were not identified in our earlier study highlights the improved capacity for identifying likely weak and transient interactions that cannot be seen in approaches based on cryogenic cell disruption. Depletion of either TbCAP80 or TbCAP141 gives strikingly similar phenotypes, suggesting related yet non-redundant functions. This is further supported by similarity in predicted secondary structure. Whilst unrelated at the sequence level to canonical monomeric clathrin adaptors, such as epsin or CALM/AP180, TbCAP80 and TbCAP141 do share a striking similarity of domain organisation, with folded N-terminal domains and disordered C-terminal regions that harbour predicted clathrin-binding motifs. This is suggestive of a degree of mechanistic similarity, and we have shown that the N-terminal folded domain of TbCAP141 carries a membrane-targeting signal which may conceivably be functionally analogous to the ENTH and ANTH domains of epsin and CALM proteins. Further support for this proposition is found in the similarity of specific defects seen in clathrin-coated pit morphology following depletion of TbCAP80, TbCAP141, TbCALM or TbEpsinR. It appears that ablation of any of these proteins leads to endocytic inhibition through a block of endocytic pit maturation, causing an accumulation of broad, flat clathrin-coated structures at the flagellar pocket. Interestingly, both whole-genome RNAi and proteomics studies indicate that TbCAP80 and TbCAP141 are also essential in the procyclic form, but are expressed at somewhat lower levels (∼0.36-fold and ∼0.25-fold, respectively, relative to in the bloodstream form), suggesting that they also have roles in endocytic transport in the insect form (Alsford et al., 2011; Urbaniak et al., 2012).

That depletion of any of the proteins that are suggested to independently drive clathrin recruitment causes malformed pits, rather than a failure of clathrin assembly per se, may be explained by an emerging model of membrane vesicle formation. In addition to proposed membrane-curvature-driving domains, such as amphipathic helices, the steric interactions between coat proteins themselves are suggested to generate positive curvature through molecular crowding (Busch et al., 2015; Copic et al., 2012; Miller et al., 2015; Stachowiak et al., 2012). The concentration of luminal cargoes packaged into forming pits generates an opposing force that must be overcome during coat assembly. *T. brucei*, with its dense VSG coat, would therefore be predicted to be particularly sensitive to perturbations of this curvature-driving force provided by molecular crowding of coat proteins. We suggest therefore that depletion of any one of the major components of the endocytic coat in *T. brucei*, while likely not sufficient to prevent clathrin assembly, leads to a failure of pit closure and subsequent endocytic inhibition.

Despite being heavily studied in yeast and metazoan systems, attention has only relatively recently turned to the degree of lineage-specific adaptation in the molecular mechanisms of CME. In contrast to the adaptin-related TPLATE/TSET complex recently characterised in plants and Amoebozoa (Gadeyne et al., 2014; Hirst et al., 2014), TbCAP80 and TbCAP141 appear to be true lineage-specific innovations. This adds to the remarkably large number of lineage-specific clathrin-associated proteins with roles in trypanosome endocytosis (Adung'a et al., 2013) and is further evidence for significant remodelling of endosomal systems to enable specific adaptation, in this instance facilitating parasitism. While less extensive than the evolution of rhoptries and dense granules as unique organelles intimately associated with cell invasion in Apicomplexa (Tomavo, 2014), it is clear that the endosomal system of trypanosomes plays vital roles in the pathogenesis of these organisms.

In summary, this study reveals the molecular pathways subtending an evolutionarily early branching and highly divergent form of CME that arose via the acquisition of a largely novel set of gene products including TbCAP80 and TbCAP141, which are conserved and likely functionally important across the kinetoplastid parasites.

## MATERIALS AND METHODS

### Cell culture and transfection

Procyclic-form (PCF) Lister 427 *Trypanosoma brucei* *brucei*, bloodstream-form (BSF) Lister 427 and single-marker bloodstream-form (SMB) parasites were cultured as previously described (Brun and Schönenberger, 1979; Hirumi and Hirumi, 1989; Wirtz et al., 1999). For generation and maintenance of lines harbouring selectable markers, antibiotics were used at the following concentrations: G418, 1 μg/ml; puromycin, 0.2 μg/ml; and hygromycin B, 5 μg/ml. COS-7 cells were cultured in Dulbecco's modified Eagle's medium (DMEM) with 10% fetal bovine serum, 100 U/ml penicillin, 100 U/ml streptomycin and 2 mM L-glutamine. For transfection of bloodstream-stage parasites, 3×10^7^–4×10^7^ cells were transfected with 10 μg of DNA using an AMAXA nucleofection system and the human T-cell nucleofection kit (Lonza). Monoclonal populations were obtained by limiting dilution. Procyclic-stage parasites were transfected by electroporation with a Bio-Rad Gene Pulser (1.5 kV, 25 μF). COS-7 cells were transfected with Fugene HD according to manufacturer's instructions.

### RNAi and endogenous-locus tagging

Procedures were carried out as described previously (Alibu et al., 2005; Manna et al., 2015; Oberholzer et al., 2006). Endogenous locus tagging cassettes were generated by PCR using the pMOTag system and gene-specific primer pairs as follows: TbCHC F, 5′-CCACCCGCAACCGGGCTACGGTGGTGTGCCCGGTCAGGGATATGCTGGAGGGATGGGAAACCCTAACATGATGCCATACGGTACCGGGCCCCCCCTCGAG-3′, TbCHC R, 5′-ATTTCTTCCCCTCCACCTACTCACCCTTTTCTCCTCCCATCTCCCTTCCCTGTGTTTCTTTTGTCCTTTTGGGCTGGCGGCCGCTCTAGAACTAGTGGAT-3′; TbCAP141 F, 5′-GAGTTCGCTCATCCTCCTGGTCGTGGTGTATGTGCTGAATGAGGGTCGTTATACACCTTTTGCCGCCCGTTTTCCAGTAGGTACCGGGCCCCCCCTCGAG-3′, TbCAP141 R, 5′-CACCAAACCAGCCTTGATATTAATTCCTTCACTTCCTCTATACGGGTTCTTCATATCACTTCCACGAATGCAAGTGGCGGCCGCTCTAGAACTAGTGGAT-3′; TbCAP80 F, 5′-CTTTGGGTCCGATCACCTCAGTGTCAGCAAAGATAAGAGGGAGAGTGGGAATCACACGCTTACTTTCAACTTTGGTAGTGGTACCGGGCCCCCCCTCGAG-3′, TbCAP80 R, 5′-AGCACATTGTTGCAGGCGTTAGAACCACTTTTTATCTCTTTCCCTTGTGTGTTTTCACTATTCTTGAAGATACCTGGCGGCCGCTCTAGAACTAGTGGAT-3′; TbSMAP F, 5′-TACTCCGAGTAATCAAGGTCCTCCGCACGTATATAGTGCTTGGGCCCCATCCGGTTCCTCCAAATGCTTTTCTCCTCAGGGTACCGGGCCCCCCCTCGAG-3′; TbSMAP R, 5′-ATTTCAGTAACTTTTTTTTGTAATTTTGCAACTTCAACTCTTTCTTCGCTAATGTAGGGCCTTCACCGCGAGTGTGGCGGCCGCTCTAGAACTAGTGGAT-3′; TbGLP1 F, 5′-TACATCTCCAACATCCCAACCGCCGGTGCGGCGGTGAACTCGGCCGGTACGAAAGGCTCCGTCATTGAGGTGGAGGATGGTACCGGGCCCCCCCTCGAG-3′, TbGLP1 R, 5′-TTTTCCTCGTTCCGGAAGGGTCCATGTTCTTCAAACACCCACTGCTACCTGGTACTCTCGTCAACCGTTCAATGGCGGCCGCTCTAGAACTAGTGGAT-3′; and TbAAK F, 5′-GGAAACGACCTATTCCAAAGGCCACAGCAGCAACAGCCACAGCAACCAGAGAAGGACCCCTTCGCCAGTCTCTTCAAGGGTACCGGGCCCCCCCTCGAG-3′, TbAAK R, 5′-GACCCCCCCTTTTTTTTTGGGGGGGGGGTGGCGTATGATGCTGTTCTGTGCCAGTATTGCAGTCATGTAAAACATGGCGGCCGCTCTAGAACTAGTGGAT-3′. Correct integration of the tagging cassette was verified by western blot. Gene-specific RNAi fragments were selected with RNAit (Redmond et al., 2014) and amplified from genomic DNA using the following primer pairs: TbAAK F, 5′-TTCTGCTTCTCGCAGACTGA-3′, TbAAK R, AAGAGGTCATCCGTTGTTGG-3′; TbCAP141 F, 5′-GCAGTTGGAGGAGCTACTGG-3′, TbCAP141 R, 5′-TTTCTCTTTCGAAGTGCGGT-3′; TbCAP80 F, 5′-CATGCGGAAAGAAAACCAAT-3′; TbCAP80 R, 5′-GCTCTTGTTTCTGTGGAGCC-3′; and TbSMAP F, 5′-CGAGGACGCAGAAAAAGAAC-3′, TbSMAP R, 5′-TGGGCAAGTACTAACCTCGG-3′. PCR fragments were cloned into p2T7^TA^blue and constructs were verified by Sanger sequencing. Multiple clonal RNAi lines were generated for each construct in the corresponding genomic-locus-tagged SMB background and assayed for robust and reproducible protein loss following tetracycline (1 μg/ml) addition by western blotting prior to phenotypic characterisation.

### Cryoimmunoisolation and proteomics

Approximately 10^10^ mid-log phase BSF or 5×10^10^ PCF parasites were harvested by centrifugation (800 ***g*** for 15 min) and washed with chilled PBS supplemented with 5.5 mM glucose. Cells were re-suspended in chilled PBS plus glucose with protease inhibitors and 10 mM dithiothrietol to a density of 5×10^9^ cells/ml and flash frozen in liquid nitrogen to preserve protein–protein interactions as close to native state as possible. Cells were cryomilled into a finely ground powder in a planetary ball mill (Retsch). For a detailed protocol refer to Obado et al. (2016a) or the National Center for Dynamic Interactome Research website (www.ncdir.org/public-resources/protocols/). Aliquots of cryo-lysate powder were re-suspended in immunoprecipitation buffer [100 mM sodium phosphate pH 7.4, 250 mM Na-citrate, 0.5% Tween 20 and protease inhibitors without EDTA (Roche)]. Subsequent affinity capture was performed as previously described using either magnetic beads coupled to anti-GFP antibody or uncoupled beads to serve as a control for non-specific interaction with the beads (Obado et al., 2016a,b). Affinity-purified proteins were eluted with 2% SDS in 40 mM Tris-HCl pH 8.0, reduced in 50 mM DTT and alkylated with 100 mM iodoacteamide prior to downstream analysis (SDS-PAGE followed by protein identification by using ESI or MALDI-TOF mass spectrometry). Eluates were fractionated on pre-cast Novex 4-12% Bis Tris gels (Life Technology), stained using colloidal Coomassie (GelCode Blue–Thermo) and analysed by mass spectrometry as previously described (DeGrasse et al., 2009).

### Immunofluorescence localisation and endocytosis assays

Parasites were cultured, harvested and prepared as described previously (Manna et al., 2015). Anti-HA (3F10, Roche) was used at 1:1000 and polyclonal rabbit anti-TbCHC (Morgan et al., 2001) was used at 1:2500. For localisation of GFP-tagged TbCAP80 and TbCAP141 truncations in bloodstream-form trypanosomes, the high-level protein expression required to image native GFP fluorescence led to mislocalisation as compared to endogenously expressed tagged proteins. Therefore, in these experiments, expression levels were restricted to near endogenous levels and the fluorescence signal was boosted by staining with polyclonal rabbit anti-GFP (1:250, ab6556; Abcam) followed by Alexa-Fluor-488-conjugated secondary antibody (Invitrogen). Wide-field fluorescence images were acquired using a Nikon Eclipse E600 microscope with a Hamamatsu ORCA CCD camera and MetaMorph software (Universal Imaging, Marlow, UK). For structured illumination microscopy (SIM), images were acquired under a 60×, 1.42 NA objective using a DeltaVision OMX V3 system (Applied Precision, Preston, UK) and deconvolved using softWoRx 5.0 software (Applied Precision). Endocytic activity was assessed by fluorescent ligand uptake over a 45 min period as described previously (Gabernet-Castello et al., 2009; Manna et al., 2015). Quantification of fluorescence intensities was carried out in Fiji software (National Institutes of Health, USA, http://fiji.sc/Fiji). Image panels were prepared in Photoshop (Adobe).

### Homology searches, domain predictions and phylogenetic analyses

For the identification of homologous sequences and reconstruction of protein phylogenies, relevant excavate sequences were first identified. To identify related proteins from the kinetoplastids, BLAST searches were carried out at TriTrypDB (http://tritrypdb.org) against *Trypanosoma vivax*, *Trypanosoma cruzi*, *Trypanosoma brucei*, *Trypanosoma grayi*, *Trypanosoma congolense*, *Leishmania seymouri*, *Leishmania tarentolae*, *Leishmania mexicana*, *Leishmania major*, *Leishmania donovani*, *Leishmania infantum*, *Crithidia fasciculate* and *Endotrypanum monterogeii*. *Bodo saltans* was searched at GeneDB (http://www.genedb.org), *Naegleria gruberi* was searched at the Joint Genome Institute (http://genome.jgi.doe.gov). Any identified excavate sequences were then used in an attempt to identify homologues from other eukaryotic supergroups. Initial protein-BLAST and protein-PSI-BLAST searches were carried out at NCBI (http://www.ncbi.nlm.nih.gov) against the non-redundant protein sequence database or species-specific protein databases where appropriate. Where no clear homologues could be identified outside of the excavates, multiple protein alignments of excavate sequences were generated and used as queries against the UniProt KB database via the Jackhmmer iterative search algorithm (http://www.ebi.ac.uk/Tools/hmmer/search/jackhmmer). Finally, all identified sequences were verified by reciprocal BLAST and/or jackhammer searches and manual inspection of alignments. To identify conserved domains, retrieved sequences were run through HHPRED (http://toolkit.tuebingen.mpg.de/hhpred), hmmscan (http://www.ebi.ac.uk/Tools/hmmer/search/hmmscan) and InterProScan (http://www.ebi.ac.uk/interpro/search/sequence-search). For phylogenetic reconstruction, sequences were aligned using MergeAlign (Collingridge and Kelly, 2012) and edited for gaps and low conservation. The correct evolutionary model was assessed using ProtTest (http://darwin.uvigo.es/software/prottest2_server.html) and phylogenetic trees were constructed using Bayesian (MrBayes, https://www.phylo.org) and maximum likelihood (PhyML, http://www.atgc-montpellier.fr/phyml/) approaches. MrBayes analyses were run using a mixed model for at least 10^6^ generations and until convergence, assessed by standard deviation of splits frequencies of <0.05.

### Mammalian expression of TbCAP141 and TbCAP80 truncations

For mammalian expression of full-length and truncated forms of TbCAP141 and TbCAP80, the coding sequences were amplified from bloodstream-form Lister 427 *Trypanosoma brucei brucei* genomic DNA and inserted into the pEGFP-N2 (Clontech) vector for C-terminal GFP tagging and mammalian expression. TbCAP141 full-length was amplified using primers: forward, 5′-GAGCTCATGCCGCTATACAATATCACTATTCA-3′ and reverse, 5′-GAATTCTACTGGAAAACGGGCGGC-3′. The N-terminal folded domain (residues 1–440) was amplified using the same forward primer and the reverse primer 5′-GAATTCGGGACCGCCGGAGGCTGCT-3′. The extreme N-terminal putative β-rich region (residues 1–165) was cloned using the same forward primer and the reverse primer 5′-GAATTCGAGGCGAACTGTTTCCACTG-3′. These PCR fragments were inserted between the SacI and EcoRI sites of pEGFPN-N2. TbCAP80 fragments were generated using the common forward primer 5′-AGATCTATGCCCGAGGCGCATATT-3′ and reverse primers 1–421, 5′-AAGCTTGAACGGGCCCATTTGAGCAC-3′ and 1–175, 5′-AAGCTTGAATCGGTAGCCATCCGCTT-3′. These fragments were cloned between BglII and HinDIII sites. For doxycycline-inducible expression in bloodstream-form trypanosomes at near-endogenous levels, the corresponding constructs were cloned into a T7 RNA polymerase-independent inducible expression plasmid as previously described (Sunter et al., 2012).

### Thin-section transmission electron microscopy

Samples were prepared as previously described (Gadelha et al., 2009). Briefly, cells were fixed isothermally in culture with 2.5% (v/v) glutaraldehyde at 37°C prior to harvesting by centrifugation (800 ***g*** for 15 min). Following fixation, samples were post fixed in 1% (w/v) osmium tetroxide in PBS for 30 min at room temperature and *en bloc* stained with 1% (w/v) aqueous uranyl acetate. Following dehydration through an acetone series, samples were embedded in epoxy resin. Ultrathin (70 nm) sections were post stained with 2% uranyl acetate and lead citrate. Samples were imaged on a Tecnai G2 transmission electron microscope (FEI, USA).
